# A real world comparison of sulfonylurea and insulin vs. incretin-based treatments in patients not controlled on prior metformin monotherapy

**DOI:** 10.1186/s12933-015-0172-9

**Published:** 2015-02-03

**Authors:** Anselm K Gitt, Peter Bramlage, Steffen Schneider, Diethelm Tschöpe

**Affiliations:** Institut für Herzinfarktforschung Ludwigshafen an der Universität Heidelberg, Bremser Strasse 79, 67063 Ludwigshafen, Germany; Herzzentrum Ludwigshafen, Medizinische Klinik B, Kardiologie, Ludwigshafen, Germany; Institut für Pharmakologie und präventive Medizin, Mahlow, Germany; Stiftung, ‘‘Der herzkranke Diabetiker’’ in der Deutschen Diabetes Stiftung, Bad Oeynhausen, Germany; Herz- und Diabeteszentrum Nordrhein- Westfalen, Universitätsklinik der Ruhr Universität Bochum, Bad Oeynhausen, Germany

**Keywords:** Diabetes, Strategies, Oral antidiabetic drugs, Insulin, Outcomes, Glucose, Effectiveness

## Abstract

**Aims:**

Metformin is the first line drug for patients diagnosed with type-2 diabetes; however, the impact of different treatment escalation strategies after metformin failure has thus far not been investigated in a real world situation. The registry described herein goes some way to clarifying treatment outcomes in such patients.

**Methods:**

DiaRegis is a multicentre registry including 3,810 patients with type-2 diabetes. For the present analysis we selected patients being treated with metformin monotherapy at baseline (n = 1,373), with the subsequent addition of incretin-based drugs (Met/Incr; n = 783), sulfonylureas (Met/SU; n = 255), or insulin (n = 220).

**Results:**

After two years 1,110 of the initial 1,373 patients had a complete follow-up (80.8%) and 726 of these were still on the initial treatment combination (65.4%). After treatment escalation, compared to Met/Incr (n = 421), Met/SU (n = 154) therapy resulted in a higher HbA1c reduction vs. baseline (−0.6 ± 1.4% vs. −0.5 ± 1.0%; p = 0.039). Insulin (n = 151) resulted in a stronger reduction in HbA1c (−0.9 ± 2.0% vs. −0.5 ± 1.0%; p = 0.003), and fasting plasma glucose (−24 ± 70 mg/dl vs. −19 ± 42 mg/dl; p = 0.001), but was associated with increased bodyweight (0.8 ± 9.0 kg vs. −1.5 ± 5.0 kg; p = 0.028). Hypoglycaemia rates (any with or without help and symptoms) were higher for patients receiving insulin (Odds Ratio [OR] 8.35; 95% Confidence Interval [CI] 4.84-14.4) and Met/SU (OR 2.70; 95% CI 1.48-4.92) versus Met/Incr. While there was little difference in event rates between Met/Incr and Met/SU, insulin was associated with higher rates of death, major cardiac and cerebrovascular events, and microvascular disease.

**Conclusions:**

Taking the results of DiaRegis into consideration it can be concluded that incretin-based treatment strategies appear to have a favourable balance between glycemic control and treatment emergent adverse effects.

**Electronic supplementary material:**

The online version of this article (doi:10.1186/s12933-015-0172-9) contains supplementary material, which is available to authorized users.

## Background

Metformin is generally the first choice antidiabetic treatment option for patients not achieving adequate blood glucose control using dietary restrictions alone [1-3]. Sulfonylurea (SU), glitazones, incretin-based treatments, and insulin are potential subsequent treatment steps according to the recent consensus statement of the European Association for the Study of Diabetes and the American Diabetes Association [3]. These are considered when monotherapy with metformin alone does not maintain HbA1c levels at target for approximately 3 months. However, actual utilisation and performance of these different strategies with respect to outcomes in real world clinical practice has not been assessed in detail.

In the present analysis we aimed to 1) describe treatment utilisation and patient characteristics of sulfonylurea (SU) and insulin vs. incretin-based treatment in a real world setting, 2) identify patients with stable treatment throughout a two year follow-up, and 3) to compare blood glucose control, body weight, rates of hypoglycaemia and incident co-morbidity/vascular events among the different treatment strategies in those with stable drug treatment.

## Methods

DiaRegis is a prospective, observational, multicentre cohort study including 3,810 patients with type-2 diabetes under the patronage of the foundation “Der herzkranke Diabetiker”, Germany. It was conducted in accordance with Good Epidemiology Practice and applicable regulatory requirements. The protocol was approved by the ethics committee of the Landesärztekammer Thüringen in Jena, Germany on March 4th 2009 and published at baseline [4]. All patients enrolled into this registry provided written informed consent and were followed for a total of 24 months.

### Patients

The principal design characteristic of DiaRegis was that consecutive patients being treated with one or two oral antidiabetic drugs were enrolled. A second criterion was that the treating physician had decided to intensify treatment at the baseline visit due to inadequate glycaemic control. Intensification was achieved by either increasing the dose of originally prescribed drugs and/or by exchanging drugs, or by prescribing additional drugs. According to protocol, there was no interaction with the physician in terms of patient selection, nor was the direction of intensification pre-defined.

Patients without treatment intensification or those on injectable antidiabetic drug therapy prior to baseline were not considered eligible. Furthermore, those not under regular supervision of the treating physician for the duration of the study, those with type-1 diabetes, pregnancy, diabetes secondary to malnutrition, infection or surgery, with maturity onset diabetes of the young, known cancer or limited life expectancy, acute emergencies, participation in another clinical trial, and patients with other reasons that would make it difficult for them to participate and attend the follow-up visits were excluded from participation.

For the present analysis patients were considered that were receiving metformin monotherapy prior to baseline, with treatment being escalated using either incretin-based drugs, i.e. dipetidyl peptidase-4 inhibitors (DPP-4 I) and glucagon-like protein-1 agonists (GLP-1 A) (Met/Incr), sulfonylureas (Met/SU), or insulin. For this purpose, only drug prescriptions, not doses, were considered. Drugs were recorded as drug classes and no doses were documented.

### Physicians

Physicians (general practitioners, internists, practitioners, and diabetologists) were selected based on a conditioned random sampling method. A physician database with approximately 9,350 office based physicians treating patients with type-2 diabetes were approached in writing, and physicians with at least 150 patients with type-2 diabetes under regular medical care and with a random distribution across all German regions were asked to participate. This resulted in 313 participating physicians, representing 3.3% of those initially approached.

### Documentation

Patient data were entered via a secure website directly into an electronic database maintained at the Institut für Herzinfarktforschung, Ludwigshafen, Germany. At this stage, they were automatically checked for plausibility and completeness. A detailed over view of the data collection procedure has been published previously [4]. Data from the patient questionnaire which each patient was asked to complete during the baseline visit were transferred to the clinical research organisation, Winicker Norimed. The questionnaires were scanned and transferred to the Institut für Herzinfarktforschung for evaluation. All data sets were checked for incorrect data and corrected if applicable; all corrections were documented. All data sets were submitted for biostatistical analysis.

Data on co-morbid disease conditions and risk factors were obtained on an anamnestic basis from the treating physician, but diagnoses were not objectively verified. Major adverse cardiac and cerebral events (MACCE) were defined as any of death, non-lethal myocardial infarction (MI), or non-lethal stroke. Microvascular complications included previously unknown retinopathy, nephropathy, neuropathy, and amputation. Macrovascular complications were defined as new MI, stroke, and peripheral artery disease (PAD) (including any peripheral intervention).

### Statistical analysis

Statistical analysis was performed using SAS, version 9.3 (Cary, North Carolina, U.S.A.). The distribution of continuous variables is described with medians and quartiles. Categorical parameters are presented as percentages and absolute numbers. All descriptive statistics are based on available cases. The adjusted prognostic values of patient characteristics, laboratory values at baseline, and co-morbidities on different events during the follow up period were investigated by using logistic regression analyses. The resulting odds ratios are presented with corresponding 95% confidence intervals (CI).

## Results

### Patient characteristics

**Figure 1:**
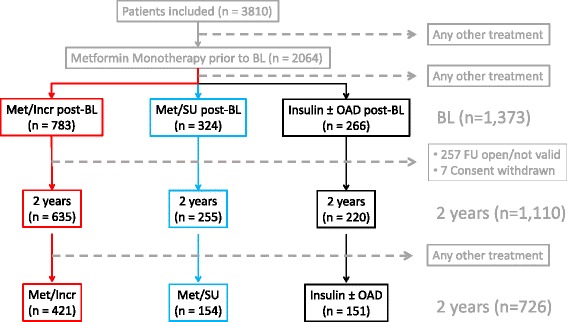
**Patient population.** Legend: Met/Incr, metformin/incretin; Met/SU, metformin/sulfonylurea; OAD, oral antidiabetic drug; BL, baseline; FU, follow-up.

A total of 3,810 patients were included in DiaRegis, of which 2,064 received metformin monotherapy at baseline (Figure 1) and 1,373 of these had an incretin-based treatment strategy (DPP-4 I or GLP-1 A) (38.0% of patients; n = 783), or SU (15.7%; n = 324), and an insulin-based treatment strategy (12.9%; n = 266) added. Other treatment options were selected in 33.4% of patients.

Patients selected for Met/Incr treatment (Table 1) had a lesser age (median 64.0 vs. 67.5 years; p = 0.026), and a higher bodyweight (median 90.0 vs. 86.0 kg; p = 0.029) than patients receiving Met/SU. Compared to insulin patients Met/Incr patients had a lesser diabetes duration (4.6 vs. 6.6 years; p < 0.001), lower rates of anamnestic hypoglycaemia (3.6 vs. 23.3%; p < 0.001), and lower blood glucose values (HbA1c 7.3 vs. 8.1%; fasting plasma glucose, FPG 137 vs. 156 mg/dl; postprandial glucose [PPG] 176 vs. 205 mg/dl). Furthermore, patients receiving insulin had a higher prevalence of microvascular disease than patients receiving Met/Incr (22.9 vs. 14.7%; p = 0.002).

**Table 1:** **Patient characteristics at baseline by treatment strategy (n = 1,373)**

	**Met/Incr**	**Met/SU**	**p-value vs. Met/Incr**	**Insulin ± any OAD**	**p-value vs. Met/Incr**
	**(n = 783)**	**(n = 324)**		**(n = 266)**	
Age (years)	64.0 (56.7–72.0)	67.5 (58.2–72.8)	0.026	62.6 (54.5–71.5)	0.074
Female gender (%)	47.6	46.9	0.83	42.1	0.12
Body weight (kg)	90.0 (79.0–103.0)	86.0 (78.0–98.5)	0.029	89.0 (78.0–100.0)	0.99
Diabetes duration (years)	4.6 (2.1–7.8)	5.2 (2.5–8.6)	0.079	6.6 (3.5–9.9)	<0.001
Blood glucose					
HbA1c (%)	7.3 (6.8–7.9)	7.3 (6.8–8.1)	0.75	8.1 (7.3–9.5)	<0.001
FPG (mg/dl)	137 (118–164)	139 (117–162)	0.91	156 (132–205)	<0.001
PPG (mg/dl)	176 (147–211)	178 (158–215)	0.13	205 (174–241)	<0.001
Anamnestic hypoglycaemia* (%)	3.4	5.2	0.16	23.3	<0.001
Concomitant disease (%)					
MACCE (%)	9.4	11.2	0.37	10.6	0.57
Macrovascular complication (%)	13.4	13.9	0.80	16.6	0.19
Microvascular complication (%)	14.7	12.4	0.31	22.9	0.002

Legend: Met/Incr, metformin/incretin; Met/SU, metformin/sulfonylurea; OAD, oral antidiabetic drug; HbA1c, glycosylated haemoglobin A1c, FPG, fasting plasma glucose; PPG, postprandial plasma glucose; MACCE, major cardiac or cerebrovascular event; *within 12 months prior to baseline.

For 1,110 out of 1,373 patients with either treatment strategy, data on the 24 month follow-up were available (635 Met/Incr, 255 Met/SU, and 220 insulin). Of these, 65.4% retained the initially chosen treatment option, while 34.6% were switched to another treatment strategy (Additional file 1: Table S1). Treatment continuity was 66.1% for Met/Incr, 60.4% for Met/SU, and 68.6% for insulin. The characteristics of patients switched to an alternative treatment versus those being stable on their baseline medication are displayed in Table 2.

**Table 2:** **Patient characteristics at baseline by treatment continuity during 24 month follow-up (n = 1,110)**

	**Met/Incr (n = 635)**		**Met/SU (n = 255)**		**Insulin ± any OAD (n = 220)**	
	**Stable**	**Switch**	**Stable**	**Switch**	**Stable**	**Switch**
	**(n = 421; 66.3%)**	**(n = 214; 33.7%)**	**(n = 154; 60.4%)**	**(n = 101; 39.6%)**	**(n = 151; 68.6%)**	**(n = 69; 31.4%)**
Age (years)	65.6 (57.7–73.0)	61.6 (55.0–69.2)	68.6 (59.6–73.2)	64.8 (56.8–71.2)	64.0 (54.6–72.9)	58.8 (54.1–65.1)
Female gender (%)	48.7	48.6	51.9	34.7	42.4	44.9
Body weight (kg)	89 (78–101)	91 (80–104)	87 (77–95)	86 (79–103)	90 (80–106)	89 (78–98)
Diabetes duration (years)	4.6 (2.1–7.6)	4.5 (1.7–7.8)	5.3 (2.4–8.6)	5.0 (2.5–8.6)	7.1 (3.6–10.7)	5.6 (2.6–8.3)
Blood glucose						
HbA1c (%)	7.2 (6.8–7.7)	7.4 (6.8–8.0)	7.1 (6.7–7.9)	7.3 (6.9–8.2)	8.3 (7.3–9.6)	7.9 (7.1–8.9)
FPG (mg/dl)	134 (116–158)	140 (120–165)	135 (114–158)	133 (117–158)	156 (130–206)	150 (123–182)
PPG (mg/dl)	174 (146–210)	178 (145–210)	177 (158–207)	169 (154–196)	202 (171–239)	198 (165–235)
Anamnestic hypoglycaemia* (%)	2.9	5.1	4.5	7.9	19.2	43.5
Concomitant disease (%)						
MACCE (%)	9.3	10.3	11.7	10.0	12.0	4.3
Macrovasc. complication (%)	12.9	16.0	14.3	14.0	20.0	8.7
Microvasc. complication (%)	12.1	18.7	10.3	13.9	23.2	11.6

Legend: Met/Incr, metformin/incretin; Met/SU, metformin/sulfonylurea; OAD, oral antidiabetic drug; HbA1c, glycosylated haemoglobin A1c, FPG, fasting plasma glucose; PPG, postprandial plasma glucose; MACCE, major cardiac or cerebrovascular event; *within 12 months prior to baseline.

### Blood glucose control and body weight in those with stable drug treatment

There was a drop in body weight after 24 months in the Met/Incr (−1.5 ± 5.0 kg). The change in body weight was not statistically different compared to Met/Incr in the Met/SU group (−0.4 ± 4.8 kg; p = 0.17) but there was an increase in body weight in the insulin group (+0.8 ± 9.0 kg; p = 0.028).

All treatment strategies were associated with reduced levels of HbA1c and FPG but an increase in posprandial glucose levels (Table 3). While changes were pronounced during the first 6 months, they were generally stable thereafter for Met/Incr- and Met/SU-treated patients, but levels increased slightly for patients using insulin. The only significant difference between Met/Incr and the Met/SU group was seen for mean HbA1c which was less reduced in the Met/Inr group (−0.5 ± 1.0%; p = 0.039). Compared to the insulin group, patients with Met/Incr had a lesser reduction of HbA1c (−0.5 ± 1.0% vs −0.9 ± 2.0%; p = 0.003) and fasting blood glucose (−19 ± 42 mg/dl vs. -24 ± 70 mg/dl; p = 0.001) while differences in mean PPG changes were not significant (27 ± 463 mg/dl vs. 90 ± 535 mg/dl; p = 0.066).

**Table 3:** **Changes in efficacy variables from baseline to 24 months for those remaining on the chosen treatment (n = 726)**

	**Met/Incr**	**Met/SU**	p-value* vs. Met/Incr	**Insulin ± any OAD**	p-value* vs. Met/Incr
	**(n = 421)**	**(n = 154)**		**(n = 151)**	
HbA1c (%)					
BL mean ± SD	7.4 ± 1.1	7.5 ± 1.4		8.6 ± 1.7	
Mean change vs. BL ± SD	−0.5 ± 1.0	−0.6 ± 1.4	0.039	−0.9 ± 2.0	0.003
FPG (mg/dl)					
BL mean ± SD	142 ± 41	143 ± 43		173 ± 66	
Mean change vs. BL ± SD	−19 ± 42	−15 ± 41	0.34	−24 ± 70	0.001
PPG (mg/dl)					
BL mean ± SD	201 ± 243	189 ± 52		224 ± 130	
Mean change vs. BL ± SD	27 ± 463	60 ± 330	0.61	90 ± 535	0.066
Body weight (kg)					
BL mean ± SD	91.2 ± 17.9	88.1 ± 14.8		93.7 ± 19.6	
Mean change vs. BL ± SD	−1.5 ± 5.0	−0.4 ± 4.8	0.17	0.8 ± 9.0	0.028

Legend: BL, baseline; Met/Incr, metformin/incretin; Met/SU, metformin/sulfonylurea; OAD, oral antidiabetic drug; HbA1c, glycosylated haemoglobin A1c, FPG, fasting plasma glucose; PPG, postprandial plasma glucose; SD, standard deviation. *Adjusted for differences in baseline characteristics: age, sex, body weight, diabetes duration, heart failure, coronary artery disease, HbA1c, FPG, and PPG.

### Rates of hypoglycaemia, and incident co-morbidity/vascular in those with stable drug treatment

Hypoglycaemia was frequently asymptomatic or at a level at which there was no requirement for help (Table 4). Incidence of any hypoglycaemia was lowest in patients receiving to Met/Incr (6.5%) and it was substantially higher in those receiving Met/SU (15.4%; OR 2.70; 95% CI 1.48–4.92) or insulin (37.1%; OR 8.35; 95% CI 4.84–14.4). Even greater elevations over Met/Incr were seen for symptomatic hypoglycaemia without the need for help (insulin OR 11.45; 95% CI 5.90–22.2 and Met/SU OR 3.13; 95% CI 1.46–6.99).

**Table 4:** **Hypoglycaemia rates and events from baseline to 24 months for those remaining on the chosen treatment (%)**

	**Met/Incr**	**Met/SU**	OR (95% CI)*vs. Met/Incr	**Insulin ± any OAD**	OR (95% CI)* vs. Met/Incr
	**(n = 421)**	**(n = 154)**		**(n = 151)**	
Hypoglycaemia with need for help	0.8	0.6	1.32 (0.12–15.0)	1.4	2.42 (0.30–19.3)
Symptomatic hypoglycaemia without need for help	4.3	10.3	3.13 (1.46–6.69)	31.3	11.45 (5.90–22.2)
Asymptomatic hypoglycaemia without need for help	3.6	10.1	2.74 (1.33–8.70)	22.9	8.33 (4.33–16.0)
Any hypoglycaemia	6.5	15.4	2.70 (1.48–4.92)	37.1	8.35 (4.84–14.4)
Death	1.7	3.2	2.11 (0.65–6.87)	7.3	4.65 (1.68–12.9)
Combined endpoints					
MACCE	2.6	5.8	2.31 (0.93–5.78)	8.0	3.08 (1.27–7.48)
Macrovascular complications	1.7	2.7	1.53 (0.43–5.44)	2.1	1.04 (0.25–4.42)
Microvascular complications^†^	7.7	6.0	0.87 (0.40–1.90)	20.7	3.84 (2.13–6.90)

Legend: Met/Incr, metformin/incretin; Met/SU, metformin/sulfonylurea; OAD, oral antidiabetic drug; MACCE, major cardiac or cerebrovascular event. ^†^Excluding those with any neuropathy and any retinopathy at baseline. *Adjusted for differences in baseline characteristics: age, sex, diabetes duration, and coronary artery disease.

Death, MACCE and macrovascular complications were lowest in patients receiving Met/Incr while microvascular complications were lowest in Met/SU patients. Albeit nominally different there was no statistically significant different in event rates compared to Met/Incr in the Met/SU group. On the other hand death (OR 4.65; 95% CI 1.68–12.9), MACCE (OR 3.08; 95% CI 1.27–7.48), and microvascular complications (OR 3.84; 95% CI 2.13–6.90) were substantially increased in those receiving insulin vs. those receiving Met/Incr.

## Discussion

Following a diagnosis of type-2 diabetes, almost all patients are initially treated with metformin. Within a few months or years, however, the majority require the addition of another treatment strategy. For a number of decades, SU and insulin have been considered to be the second line treatment options, although they are associated with a progressive decline in beta-cell function. Furthermore, both are associated with a high prevalence of hypoglycaemia, weight gain, and other side effects related to individual agents [5,6]. They have also been shown to be linked with an increased incidence of cardiovascular events [6,7].

Incretin-based treatment strategies on the other hand, have been shown to preserve beta-cell function, support weight neutrality or even weight loss, and have a low intrinsic risk of hypoglycaemia. In addition gliptins have been shown to reverse pro-angiogenic cells dysfunction associated with type-2-diabetes in vitro and to improve inducible angiogenesis by circulating cells in vivo [8], normalize retinal capillary flow and improves central hemodynamics [9], and restore vascular mitochondrial adaptation to exercise in a diabetic rodent model and may augment the impact of exercise on the vasculature [10]. Most importantly, clinical trials such as EXAMINE and SAVOR TIMI 53 have shown that alogliptin as well as saxagliptin are non-inferior to a placebo in patients at high cardiovascular risk, even after acute coronary syndromes [11,12]. A recent pooled analysis of 20 clinical trials confirmed the safety for the cardiovascular system [13]. A potential limitation of the good risk profile of gliptins are the finding of an increased risk for heart failure, particularly in patients with elevated natriuretic peptides, previous heart failure or chronic kidney disease [11]. Despite their remarkably increased use, health authorities in different countries, including Germany, regard incretin-based treatment strategies to be second to those based on SU or insulin. This has prompted us to take a closer look at real world treatment patterns, treatment durability, glucose control, and other outcomes in clinical practice.

### SU vs. incretin-based treatment strategies on top of metformin

Using a large dataset of type-2 diabetic patients in Germany we were able to assess real world treatment patterns and outcomes over 24 months. A preference for adding SU in patients with higher age and low bodyweight was identified. Met/SU treatment resulted in adequate glucose control and was continued in approximately 60% of patients over the follow-up period, which was only slightly poorer than with the other treatment strategies. There was no issue with regard to weight gain, but the rates of hypoglycaemia (without the need for help) were increased with an OR of around 3. Furthermore, although nominally increased, the rate of cardiovascular events was not significantly different from those experienced by patients being treated with incretin-based therapies.

These results are principally in agreement with a recent meta-analysis regarding the effects of DPP-4 inhibitors [14], which demonstrated a slight advantage of SU over DPP-4 inhibitors in reducing HbA1c (weighted mean difference 0.07%). Furthermore, the meta-analysis identified a mean difference in body weight of −1.92 kg in favour of DPP-4 inhibitors, which was larger than the −1.4 kg we observed; however, the difference between the two treatment strategies in the present study was not statistically significant. In line with our own results, most trials comparing a DPP-4 inhibitor with SU, in combination with metformin, demonstrate a slightly higher risk of hypoglycaemia in the latter group [15-18]. Within this context, different generations of SU have to be considered, although we did not capture them individually. Second generation glibenclamide and glipizide are extensively used and are known to have a quite significant risk of hypoglycaemia [19,20]. Episodes with these agents may be persistent because of their prolonged half-life and binding to the SU receptor, causing it to be less sensitive to glucose levels [21,22]. Insulin is secreted in turn, increasing the risk of hypoglycaemia [23]. On the other hand, the third generation SUs gliclazide and glimepiride have different receptor binding properties, reducing the risk of hypoglycaemia and are potentially more favourable treatment alternatives [24,25].

Information on the risk of cardiovascular events with incretin-based treatment strategies and SU are conflicting [26,27]. Recent analyses have suggested an increased risk of death due to cardiovascular causes, with an OR between 1.92 and 2.93 in patients receiving SU therapy compared to other treatment options [26]. Furthermore, in a review of 115 randomised, controlled trials, SU was associated with an increased mortality (OR 1.22), stroke (OR 1.28), and MACCE (OR 1.85, if only the comparison with DPP-4 inhibitors was considered) [27]. On the other hand, the recent analysis by Karagiannis [14] demonstrated no difference in all-cause mortality between DPP-4 inhibitors and those receiving SU. Against this background, our finding of a nominally increased event risk with no statistical significance after multivariable adjustment appears reasonable, but does not add clarity to the ongoing dispute. Within this context, the limitations of an observational study that has a less than perfect degree of follow-up (80% in the case of DiaRegis) has to be kept in mind, given that a loss to follow-up can potentially also mean patient death.

### Insulin vs. incretin-based treatment in combination with metformin

In the present registry, insulin was predominantly used in patients with long-standing diabetes, higher blood glucose values, and a higher prevalence of microvascular disease, indicating a more advanced disease stage. Hypoglycaemia was more frequent in patients on a later insulin treatment giving rise to the speculation that these may have been patients with more advance disease, a high variability in blood glucose and a higher cardiovascular disease burden – conditions that have been associated with a higher risk of hypoglycaemia. Surprisingly, even hypoglycaemia on prior oral therapy did not prevent physicians from introducing an insulin-based treatment strategy. Insulin use was continued in 68.6% of patients throughout the 2 year follow-up. While insulin use was associated with a strong reduction in HbA1c and FPG, albeit from a higher baseline value, it was associated with increases in body weight and rate of hypoglycaemia. By the end of the two year follow-up period, 7.3% of patients had died (OR 4.65), 8.0% had experienced a MACCE (OR 3.08), and 20.7% any microvascular complication (OR 3.94), which was significantly higher than for the patients that had received an incretin-based treatment strategy in combination with metformin.

A number of considerations have to be taken into account when discussing these observations. Patients being considered for insulin treatment are usually those with an advanced stage of diabetes (median duration 6.6 vs. 4.6 years in DiaRegis) and higher blood glucose levels (HbA1c 8.1 vs. 7.3%), with corresponding changes in FPG and PPG. This is because insulin is known to effectively control hyperglycaemia and glucotoxicity, and to reduce lipotoxicity and inflammation [28]. This could potentially favourably influence the preservation of beta-cell function, but has to be prescribed at an early stage after diagnosis [29], where adverse cardiovascular effects are comparable to those experienced with other treatment options. Clinical practice surveys such as DiaRegis, however, reconfirm that insulin is usually initiated in those failing on a number of previously attempted treatment options and in patients with a high degree of co-morbidity.

Hypoglycaemia is one of the most critical factors in the management of type-2 diabetes. Insulin often causes significant hypoglycaemia in addition to weight gain, and clinical trials such as ACCORD, ADVANCE, and VADT have suggested that mortality rates may be increased in patients with episodes of severe hypoglycaemia [30-32]. Against this background, the high rates of hypoglycaemia observed in patients treated with insulin in comparison to those administered incretin-based therapies in the DiaRegis study are noteworthy, with an OR of 11.45 for symptomatic hypoglycaemia and 8.33 for asymptomatic hypoglycaemia. Rates of severe hypoglycaemia necessitating help were also increased (OR 2.42) but did not reach statistical significance.

Whether or not the previously discussed changes directly result in increased morbidity and mortality cannot be derived from the present analysis. It is evident, however, that patients selected for insulin treatment at baseline had a substantially increased death rate at 2 years (7.3 vs. 1.7%; OR 4.65), a higher rate of MACCE (8.0 vs. 2.6%; OR 3.08), and a greater incidence of microvascular morbidity (20.7 vs. 7.7%; OR 3.84). Randomised controlled studies comparing incretin-based treatments and insulin are usually too short to assess differences in outcomes; therefore it is clear that future studies should address this. In the recent DURATION-2 trial, once-weekly administration of the GLP-1 agonist exenatide was compared to insulin glargine for a total duration of 26 weeks [33]. The trial demonstrated that exenatide is a viable and more convenient alternative in those with a risk of hypoglycaemia and weight gain, but did not show differences in survival. Retrospective analyses of the United Kingdom General Practitioners research database, however, confirmed our observation that insulin treated patients had an increased risk of death (OR 2.2), MACCE (OR 1.7), and microvascular complications combined (OR 1.4) [6]. This difference may relate to the higher baseline risk noted for patients chosen for insulin over incretin-based treatments in DiaRegis.

### Limitations

Despite the real world nature of DiaRegis and the direct impact for clinical practice, there are a number of limitations that are worth mentioning: 1) We documented drug classes (yes/no) rather than specific drugs in a given class and no information on drug doses prescribed. This was done in an effort to maintain a pragmatic case report form with variables limited to the ones needed. Furthermore the recording of drugs and doses would have resulted in groups that would have been considerably smaller with no added value given the overall sample size pursued. 2) Group assignment was made based on the treatment decision made by the treating physician. On the one hand this is a limitation of the present analysis because it does not allow random group assignment and thus a comparability of groups that is required to directly compare outcomes. On the other hand this approach allows to record treatment decisions and the underlying patient characteristics being associated to these decisions. To overcome this limitation we adjusted the outcome variables for baseline characteristics, acknowledging that this might not be able to balance the groups completely. For further more elaborate analyses such as propensity score matching group size was not large enough. Finally we had a loss of about 20% of patients during the 2-year follow-up which results in some uncertainty as to the findings of this registry.

## Conclusions

Taking the results of DiaRegis into consideration, it can be concluded that incretin-based treatment strategies appear to have a favourable balance between glycemic control and treatment emergent adverse effects. in type-2 diabetics failing on metformin. The establishment of this registry has gone some way to assessing the outcomes of treatment strategies for such patients in a real world clinical setting.

## Additional file

Additional file 1: TableS1. Pharmacotherapy post baseline and after 24 months by treatment continuity.

## Abbreviations

DiaRegis: Diabetes treatment patterns and goal achievement in primary diabetes care
Met: Metformin
SU: Sulfonylurea
Incr: Incretin
HbA1c: Glycosylated haemoglobin A1c
OR: Odds ratio
CI: Confidence interval
DPP-4 I: Dipetidyl peptidase-4 inhibitors
GLP-1 A: Glucagon-like protein-1 agonists
MACCE: Major adverse cardiac and cerebral events
MI: Myocardial infarction
PAD: Peripheral artery disease
SAS: Statistical analyses system
FPG: Fasting plasma glucose
PPG: Postprandial glucose
EXAMINE: Examination of Cardiovasccular Outcomes with alogliptin versus standard of care in patients with type 2 diabetes mellitus and acute coronary syndrome
SAVOR TIMI 53: Saxagliptin assessment of vascular outcomes in patients with diabetes mellitus – thrombolysis in myocardial Infarction 53
ACCORD: Action to control cardiovascular in diabetes
ADVANCE: Action in diabetes and vascular disease: PreterAx and diamicron MR controlled evaluation
VADT: Veterans Affairs Diabetes Trial
DURATION: Diabetes Therapy Utilization: Researching changes in A1c, weight and other factors through intervention with exenatide once weekly
