# Supplementary material for: A real world comparison of sulfonylurea and insulin vs. incretin-based treatments in patients not controlled on prior metformin monotherapy
Source: Cardiovasc Diabetol. 2015 Feb 3;14:13. doi: 10.1186/s12933-015-0172-9 (PMC4324641; doi:10.1186/s12933-015-0172-9)
Supplement: Additional file 1: TableS1. — Pharmacotherapy post baseline and after 24 months by treatment continuity. [file 12933_2015_172_MOESM1_ESM.docx]

# Additional files1: Table S1 - Pharmacotherapy post baseline and after 24 months by treatment continuity

| Treatment | Metformin/Incretin (%) | | | Metformin/SU (%) | | | Insulin ± OAD (%) | | |
| --- | --- | --- | --- | --- | --- | --- | --- | --- | --- |
|  | postBL  (n=635) | Stable  (n=421) | Switch  (n=214) | postBL  (n=255) | Stable  (n=154) | Switch  (n=101) | postBL  (n=220) | Stable  (n=151) | Switch  (n=69) |
| Metformin | 100 | 100 | 81.3 | 100 | 100 | 80.2 | 78.1 | 74.3 | 92.6 |
| Sulfonylurea (SU) | 0.0 | 0.2 | 13.6 | 100 | 100 | 27.7 | 0.7 | 1.4 | 2.9 |
| Glucosidase Inhibitor | -- | -- | 0.5 | -- | -- | 1.0 | 0.7 | 0.0 | 0 |
| Glinide | -- | -- | 3.7 | -- | -- | 3.0 | 2.0 | 1.4 | 0 |
| Glitazone | -- | -- | 2.3 | -- | -- | 1.0 | 2.6 | 0.7 | 0 |
| DPP-4 I | 100 | 99.8 | 42.5 | -- | -- | 32.7 | 3.3 | 8.6 | 69.1 |
| GLP-1A | 0.0 | 0.2 | 8.4 | -- | -- | 3.0 | 0.7 | 0.0 | 1.5 |
| Insulin | -- | -- | 25.7 | -- | -- | 21.8 | 100 | 100 | 10.3 |
| Short-acting only | -- | -- | 1.4 | -- | -- | 0 | 15.9 | 10.7 | 1.5 |
| Long-acting only | -- | -- | 15.4 | -- | -- | 13.9 | 39.1 | 35.0 | 0 |
| Short / long acting | -- | -- | 6.1 | -- | -- | 6.9 | 31.1 | 40.0 | 7.4 |
| Mixed insulin | -- | -- | 2.8 | -- | -- | 1.0 | 13.9 | 14.3 | 1.5 |

Legend: postBL, post baseline; SU, sulfonylurea; OAD, oral antidiabetic drug; DPP-4 I, dipeptidylpeptidase 4 inhibitor; GLP-1 A, glucagon-like peptide 1 analogue
